# Nanomaterials for the Photothermal Killing of Bacteria

**DOI:** 10.3390/nano10061123

**Published:** 2020-06-06

**Authors:** Sibidou Yougbaré, Chinmaya Mutalik, Dyah Ika Krisnawati, Heny Kristanto, Achmad Jazidie, Mohammad Nuh, Tsai-Mu Cheng, Tsung-Rong Kuo

**Affiliations:** 1 International Ph.D. Program in Biomedical Engineering, College of Biomedical Engineering, Taipei Medical University, Taipei 11031, Taiwan; d845107003@tmu.edu.tw (S.Y.); cmutalik41@gmail.com (C.M.); 2 Institut de Recherche en Sciences de la Santé (IRSS-DRCO)/Nanoro, 03 B.P 7192, Ouagadougou 03, Burkina Faso; 3 Dharma Husada Nursing Academy, Kediri, East Java 64114, Indonesia; dyahkrisna77@gmail.com (D.I.K.); or henykristanto1@gmail.com (H.K.); 4 Department of Electrical Engineering, Institut Teknologi Sepuluh Nopember, Surabaya 60111, Indonesia; rektor@unusa.ac.id; 5 Universitas Nahdlatul Ulama Surabaya, Surabaya 60111, Indonesia; 6 Department of Biomedical Engineering, Institut Teknologi Sepuluh Nopember, Surabaya 60111, Indonesia; nuh@ee.its.ac.id or; 7 Graduate Institute of Translational Medicine, College of Medicine and Technology, Taipei Medical University, Taipei 11031, Taiwan; 8 TMU Research Center of Cancer Translational Medicine, Taipei Medical University, Taipei 11031, Taiwan; 9 Graduate Institute of Nanomedicine and Medical Engineering, College of Biomedical Engineering, Taipei Medical University, Taipei 11031, Taiwan

**Keywords:** nanomaterial, photothermal, antibacterial activity, metal nanostructure, carbon-based nanocomposite, polymer

## Abstract

An upsurge in the multidrug-resistant (MDR) bacterial pestilence is a global cause for concern in terms of human health. Lately, nanomaterials with photothermal effects have assisted in the efficient killing of MDR bacteria, attributable to their uncommon plasmonic, photocatalytic, and structural properties. Examinations of substantial amounts of photothermally enabled nanomaterials have shown bactericidal effects in an optimized time under near-infrared (NIR) light irradiation. In this review, we have compiled recent advances in photothermally enabled nanomaterials for antibacterial activities and their mechanisms. Photothermally enabled nanomaterials are classified into three groups, including metal-, carbon-, and polymer-based nanomaterials. Based on substantial accomplishments with photothermally enabled nanomaterials, we have inferred current trends and their prospective clinical applications.

## 1. Introduction

Bacterial infections constitute a serious threat to public health and remain a challenge for researchers. Despite the magnitude of antimicrobial resistance, the development and approval of new antibiotics are rare nowadays [1,2,3]. The use of antibiotics is a common way to treat infections, but this can lead to resistant bacterial strains due to mutations [4]. This situation occurs because of inappropriate uses of antibiotics. In terms of inappropriate uses, more than 90% of antibiotics were found to have been overused or misused [5]. Some situations involving surgical acts can increase healthcare costs and patient sufferance, with long-lasting antibiotherapy [6]. The long-term use of antibiotherapy, however, can also cause discomfort and toxicity.

To overcome these issues, light-based treatments that utilize nanomaterials and their composites have been developed. Among these nanomaterials, photothermal therapy (PTT) presents multiple advantages, such as being minimally invasive, remotely controllable, and efficient [7,8]. Moreover, PTT does not cause mutations in bacteria and also has a wide antibacterial spectrum [9]. Nanomaterials used for PTT in bacterial infections can be grouped into three sections: metal nanomaterials, carbon-based nanocomposites, and polymers. Organic nanoparticles (NPs) have shown excellent biocompatibility and are cheaper [10,11,12,13,14]. Metal NPs possess five-times the absorption compared to organic ones, and much smaller amounts are thus needed [15]. For many reasons, such as bacterial targeting and synergistic effects when utilizing heat and other bacterial toxicities, many metal nanocomposites have been prepared and their photothermal performances have been calibrated [16,17,18,19,20]. Another important group of photothermal agents comprises carbon-based nanomaterials (CBNs). In this group, carbon nanotubes (CNs), graphene oxide (GO), and graphene quantum dots (GQDs) have shown great potential. In addition to the aforementioned groups, polymeric nanomaterials have already been used for wound dressings due to their multiple advantages. Among them, hydrogels are the most popular for biomedical applications [21]. Therefore, nanomaterials can generally be integrated into hydrogels for PTT, especially for treating bacterial infections. In this review, we summarize recent work, presenting an explanation of the synthesis principles and antibacterial mechanisms of nanomaterials and providing relevant results. After that, directives are also proposed according to current trends.

## 2. Metal-Based Nanomaterials

**Figure 1:**
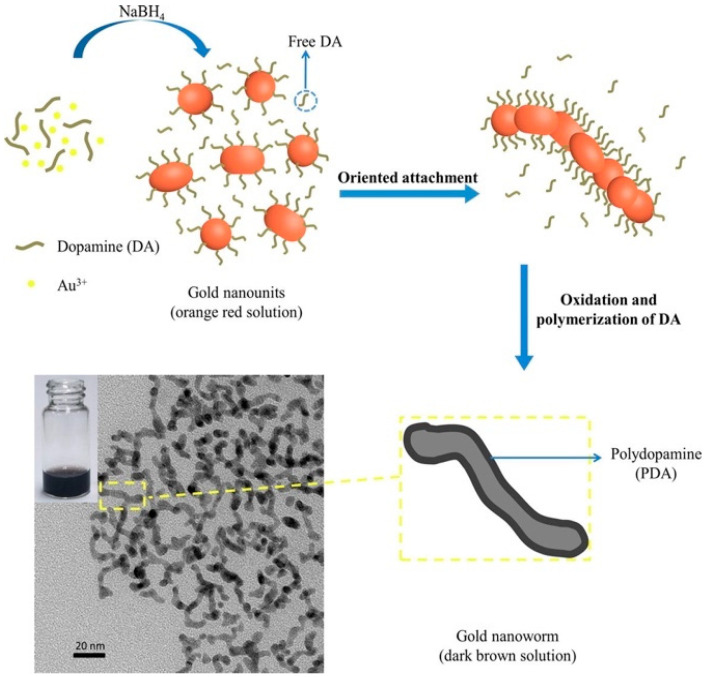
The proposed synthetic mechanism for the formation of gold nanoworms (AuNWs). Reproduced from [39], with permission from Elsevier, 2020.

Metal-based nanomaterials have received great interest due to their unique optical properties, which are size- and shape-dependent [22,23,24,25]. Therefore, many kinds of metal nanostructures have been produced, such as nanorods [26,27,28], nanostars [29], nanobipyramids [30], nanowires [31,32,33], nanoworms (NWs) [34,35,36], nanoflowers [37,38], etc. Liao et al. focused on gold NWs (AuNWs) and synthesized them with an average diameter of 5 ± 1.5 nm through a one-step synthesis method, as shown in Figure 1. After near-infrared (NIR) laser (808 nm) irradiation for 20 min, AuNW solutions of 100 and 25 μg/mL induced increases in temperature of 30.9 and 14.2 °C, respectively. After confirming the photothermal performance, the photothermal toxicities of AuNWs were evaluated through incubation with *Escherichia coli* (*E. coli*) and *Staphylococcus aureus* (*S. aureus*) using a plate counting method under a 1 W/cm^2^ intensity of NIR laser irradiation. At the end of seven cycles of NIR laser irradiation, the photothermal stability of AuNWs had improved. With NIR irradiation, AuNWs revealed antibacterial activities of >80% and 90% for *E. coli* and *S. aureus*, respectively. However, without NIR laser irradiation, AuNWs only showed antibacterial activities of <20% for both *E. coli* and *S. aureus* [39].

**Figure 2:**
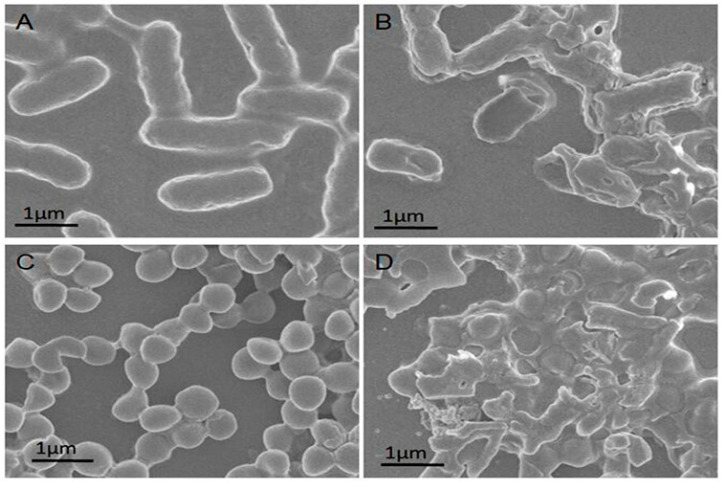
SEM images of (**A**) untreated *Escherichia coli,* (**B**) *E. coli* treated with protease-conjugated gold nanorods (PGs) (50 μg/mL), (**C**) untreated *Staphylococcus aureus,* and (**D**) *S. aureus* incubated with PGs (50 μg/mL) and near infrared (NIR) illumination for 20 min. Reproduced from [40], with permission from Medical Press, 2019.

Endotoxins secreted by certain bacteria can cause severe infections. Naturally produced biofilm also protects bacteria and increases the resistance to antimicrobial treatments. Therefore, designing materials capable of inhibiting endotoxins and biofilm formation or destroying them is a good way to fight superbugs. Li et al. fabricated protease-conjugated gold nanorods (PGs) for bacterial exotoxins and biofilm elimination under light illumination [40]. According to their work, PGs caused the degradation of nucleic acids and proteins of *E. coli* and *S. aureus* after NIR irradiation for 20 min (Figure 2). Hyperthermia generated by gold nanorods and protease activity induced the breakage of bacterial membranes and allowed the degradation of proteins and nucleic acids. Autoinducing peptide (AIP) plays an essential role in quorum sensing and was degraded by PGs. Therefore, inhibition of the AIP affects biofilm formation and broadens bacterial resistance. Their results also showed that PGs combined with NIR irradiation induced endotoxin destruction which was better than that of hyperthermia or protease alone.

Molybdenum disulfide (MoS_2_) nanosheets (NSs) are known to have an excellent photothermal performance and are capable of being functionalized by multiple biomolecules, such as poly(ethylene glycol) (PEG)-SH and immunoglobulin (Ig), and maintaining their properties. However, these conjugates are unstable in physiological solutions. Therefore, dopamine is used as an interface to assist with strong binding between MoS_2_ NSs and PEG-SH or IgG specific to the surface proteins of *S. aureus.* Synthesized nanocomposites are expected to exhibit a targeting ability, bacterial photothermal killing, and compatibility with surrounding cells of *S. aureus*. In the photothermal process, heat can affect surrounding healthy cells. Additionally, Yuwen et al. revealed the capability of biofilms to withstand heat or disturb heat dissipation [41]. To overcome these issues, metals with known photothermal effects were combined with organic compounds with specific properties, such as an affinity towards bacteria. Following this logic, one of the aims is to ensure that photothermally activated nanomaterials are situated close to the target bacterial cells. Therefore, Zhang and coworkers combined MoS_2_ silver nanocomposites with polydopamine (PDA). This MoS_2_-coated PDA was again coupled with PEG-SH and IgG of anti-protein A. The final nanocomposite was named MPPI NSs [42]. Concerning the capability of MPPI NSs to target *S. aureus* and *Pseudomonas aeruginosa,* biofilms were separately incubated with saline, MoS_2_@PDA-PEG (MPP), and MPP/IgG (MPPI) solutions for 6 h. After incubation, images from scanning electron microscopy (SEM) allowed examination. In MPP NS solutions, SEM images showed an accumulation of crumpled MPP NSs on the biofilm surface due to a lack of specific binding. However, images from MPPI NS solutions displayed a crumpled sail of NSs covering bacterial cells in the biofilm. Energy-dispersive x-ray spectroscopy (EDS) also demonstrated a greater accumulation of MPPI NSs than MPP NSs on *S. aureus* biofilm through Mo and S percentages. The targeting ability of MPPI NSs was confirmed through the results of a differential test with *P. pseudomonas*. To investigate bacterial photothermal killing by MPPI NSs, *S. aureus* biofilms were incubated with MPPI NSs and MPP NSs and irradiated with a 785-nm laser (at 0.58 W/cm^2^) for 10 min.

**Figure 3:**
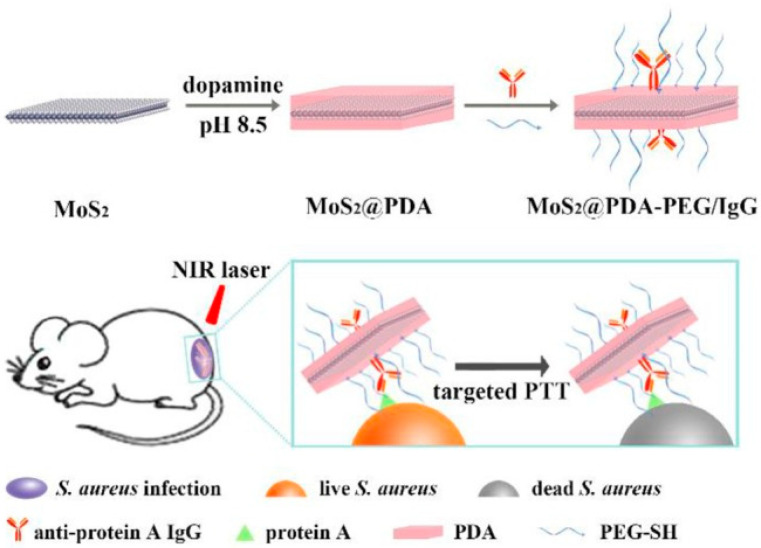
Preparation of MoS_2_@PDA-PEG/IgG nanosheets (NSs) (MPPI NSs) and their application for the targeted photothermal therapy (PTT) of *S. aureus* focal infection. Reproduced from [42], with permission from Frontiers Media S.A., 2019.

Temperatures reached 30 and 43 °C for MPP NS-biofilm and MPPI NS-biofilm blends, respectively. This showed that more MPPI NSs than MPP NSs had accumulated on the biofilm through specific binding to the antibody. Without laser irradiation, MPP NSs and MPPI NSs decreased the number of colony-forming units (CFU) on *S. aureus* biofilms by 57.08% and 77.07%, respectively. However, after irradiation, this dropped to 89.14% for MPP NSs and >99.99% for MPPI NSs at a concentration of 160 μg/mL. This confirmed the effectiveness of targeted PTT. The in vivo photothermal efficacy was also evaluated with mice wound infection observations carried out for 8 days (Figure 3). The numbers of *S. aureus* colonies in the wound were found to be more than 99.99% for MPPI NSs and 48.43% for MPP NSs after irradiation.

**Figure 4:**
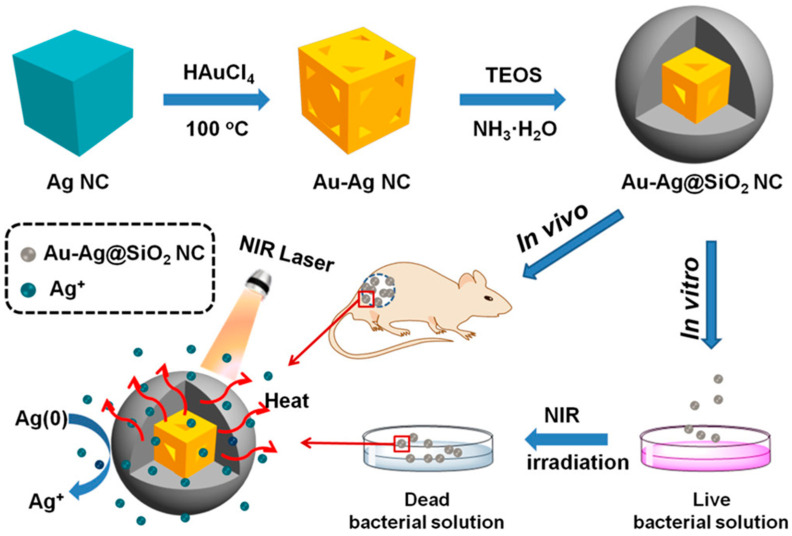
Silica-coated gold-silver nanocages (Au-Ag NCs) showing antibacterial activity by a photothermal effect. Reproduced from [46], with permission from American Chemical Society, 2019.

Gold-silver nanostructures were found to possess plasmonic resonance in the NIR region (~800 nm), making them effective photothermal candidates. Gold-silver bimetallic nanocomposites conjugated with aspartame were found to be effective antimicrobial agents under 808-nm laser irradiation. The macrophage-membrane@gold-silver nanocages were found to have enhanced microbial inhibition under laser irradiation with a specific bacterial targeting ability [43,44,45]. The recently reported novel nanomaterial silica-coated gold-silver nanocages (Au-Ag@SiO_2_ NCs) showed reliable increases in microbial resistance under NIR laser irradiation compared to Au-Ag NCs alone [46]. The surface plasmon resonance of Au-Ag NCs was improved by coating them with silicon dioxide at 770~804 nm, which is nearly complimentary to the NIR region. When synthesized Au-Ag@SiO_2_ NCs were subjected to an NIR laser at a concentration of 400 μg/mL in aqueous solution for 10 min, there was a swift increase in temperature from 20.7 to 60.1 °C. Increases in the concentration of Au-Ag@SiO_2_ NCs and the time of laser irradiation were directly proportional to an increase in temperature. This showed that the heat generated by this nanomaterial was rapid and able to eradicate *Enterococcus faecium*, *Staphylococcus aureus*, *Klebsiella pneumoniae*, *Acinetobacter baumannii*, *Pseudomonas aeruginosa*, and *Enterobacter* spp. (ESKAPE) pathogens (Figure 4) [47].

In vitro and in vivo studies have also shown an effective inhibition of bacteria under NIR laser irradiation. A silica coating of Au-Ag NCs not only enhanced the optical properties, but also assisted with the controlled release of silver ions for a prolonged period of time and showed enhanced antibacterial activity. The minimum bactericidal concentration (MBC) for *S. aureus* was found to be 128 μg/mL with an incubation period of 12 h and NIR laser irradiation (at an intensity of 1.5 W/cm^2^) for 5 min. The MBC for *E. coli* was 8 μg/mL under the same conditions as in previous studies. The photothermal killing of microbes was also evident for *S. aureus* biofilms, as confirmed by three-dimensional (3D) confocal laser microscopy (CLM). In vivo studies have reported the combined anti-infective effects of microbial growth inhibition and speedy wound healing in 7 days under NIR laser irradiation [48].

**Figure 5:**
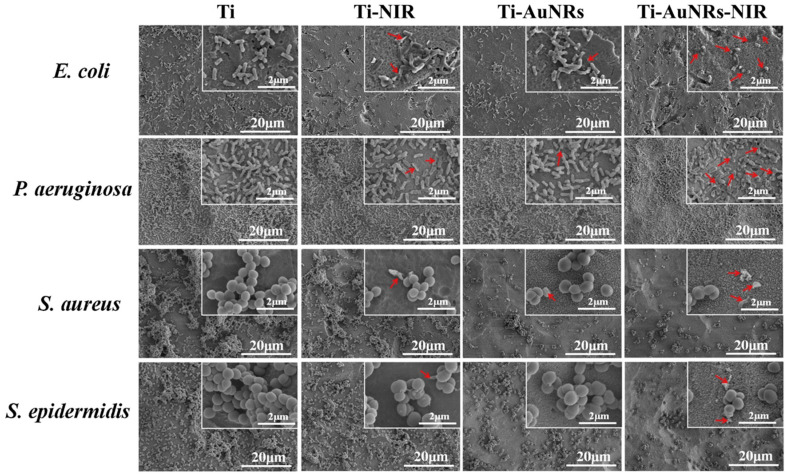
SEM images of *E. coli*, *Pseudomonas aeruginosa*, *S. aureus,* and *S. epidermidis* on titanium (Ti) and Ti-gold nanorod (Ti-AuNRs) surfaces before and after NIR irradiation (20 min); the bacteria concentration was 10^7^ colony-forming units/mL. Red arrows show the damaged microbial cell structure. Reproduced from [52], with permission from Elsevier, 2019.

Ag-doped TiO_2_ exhibited enhanced antibacterial activity against gram-positive bacteria (GPB) and gram-negative bacteria (GNB) compared to crude titanium dioxide under visible light irradiation [49]. The antibacterial activity displayed by Zn-doped titanium dioxide thin films was also notable compared to TiO_2_ or ZnO alone under the influence of visible and NIR light irradiation [50]. Au-TiO_2_ on a graphene monolayer (GTA) exhibited significant bactericidal effects compared to its counterparts of TiO_2_, graphene, and a TiO_2_-graphene layer under the influence of light irradiation [51]. One recent study showed that gold nanorods (AuNRs) coated on the surface of titanium displayed microbial resistance against the gram-negative bacteria, *E. coli* and *Pseudomonas aeruginosa* (*P. aeruginosa*)*,* with an enhanced photothermal effect by NIR irradiation and a high cell viability. Titanium in the present scenario is important in implants for clinical applications, and AuNRs on the titanium surface were found to be very effective against GPB and GNB [52]. The antimicrobial efficiencies of Ti-AuNRs towards *E. coli*, *P. aeruginosa*, *S. aureus,* and *Staphylococcus epidermidis* (*S. epidermidis*) under NIR laser irradiation were 61.82%, 66.74%, 26.31%, and 31.84%, respectively. The results indicated that the gram-negative bacilli, *E. coli* and *P. aeruginosa,* demonstrated a higher efficiency compared to two other gram-positive *Coccus* counterparts (Figure 5). The surface area, size, and shape of the bacteria are also important factors to be considered, and in this case, the bacillus, rod-shaped bacterium complimented the shape of AuNRs, and AuNRs were more effective against rod-shaped bacilli. AuNRs are less effective against coccus-shaped bacteria, as there is more gluing with rod-shaped bacilli compared to cocci. It was proven that the light absorbed by AuNPs can be transformed into heat, leading to the degradation of bacterial cell walls. The antimicrobial activity displayed by photothermal microbial treatment is more extensively used and a more notable technique, especially AuNRs, which are more capable candidates with NIR laser irradiation [52,53,54,55,56,57,58].

**Figure 6:**
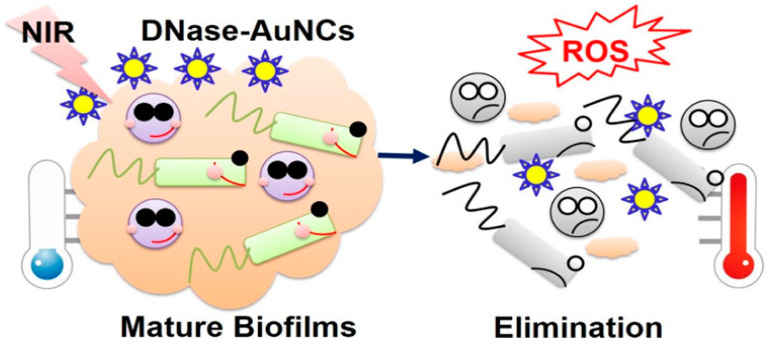
Mechanism of biofilm elimination by gold nanoclusters coated with DNase (DNase-AuNCs) under NIR irradiation. Reproduced from [59], with permission from American Chemical Society, 2020.

Gold nanoclusters coated with DNase (DNase-AuNCs) fabricated by Xie et al. showed the capability to kill both GPB and GNB. The DNase-AuNCs were toxic to planktonic bacteria and biofilms. Bacterial extracellular matrix was broken up by DNase, exposing bacterial strains to reactive oxygen species (ROS) and the heat from gold nanoclusters after NIR 808-nm laser irradiation (2 W/cm^2^) for 10 min [59]. With 800 μg/mL of gold nanoclusters, the temperature of DNase-AuNCs reached 63 °C under 808-nm laser illumination for 10 min. Using DNase-AuNCs, bacterial inhibition ratios were 35% and 60% due to the ROS and heat generated under light irradiation, respectively. Moreover, the combined bacterial inhibition ratio was 90%, which confirmed that DNase-AuNCs furnish good photothermal and photodynamic synergistic effects upon NIR illumination (Figure 6).

## 3. Carbon-Based Nanomaterials

**Figure 7:**
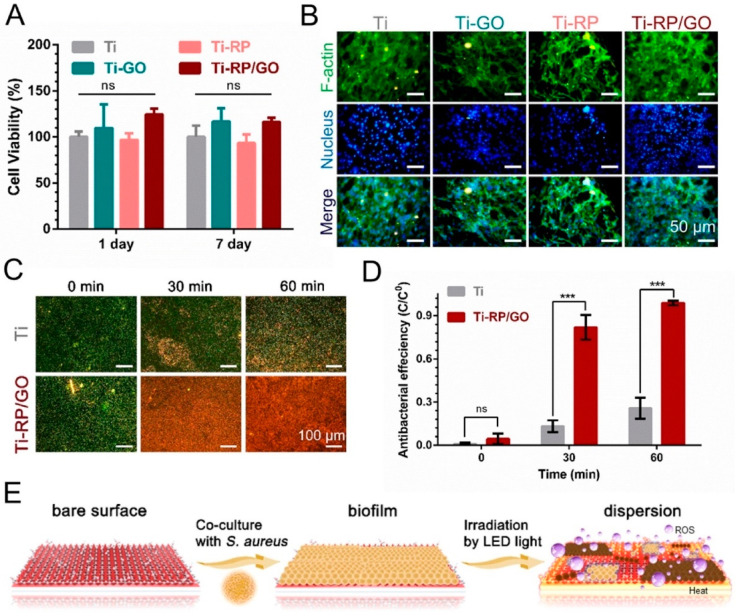
Cellular response to (**A**) the cytotoxicity of NH3T3 cells after co-culturing for 1 and 7 days and (**B**) fluorescence staining on the surfaces of samples after co-culturing with NH3T3 cells for 24 h. (**C**) The fluorescent assay of *Staphylococcus aureus* biofilm after irradiation with LED light (at 0.1 W/cm^2^). Green represents living bacteria, while red represents dead bacteria. (**D**) Quantitative analysis of the fluorescent assay by Image J software. (**E**) Schematic diagram of biofilm dispersion. Reproduced from [60], with permission from Elsevier, 2020.

Among the carbon-based nanomaterials previously listed, graphene oxide (GO), one of the carbon derivatives, possesses photothermal properties. Recently, phosphorous has aroused great interest due to its wide availability and eco-friendly properties. Amorphous red phosphorous (RP) has also been explored for bacterial inactivation. According to the advantages of RP, which is capable of absorbing NIR light, Zhang et al. fabricated a phosphorous film deposited on a Ti plate (Ti-RP). Moreover, the photoelectrochemical performances were improved by a GO layer coated onto the film. The final nanocomposite was called RP/GO film [60]. *E. coli* and *S. aureus* at 10^7^ CFU/mL mixed with RG/GO were about 99.9% inactivated under irradiation with simulated sunlight (SSL) and NIR light for 20 min at 0.2 or 0.6 W/cm^2^. Irradiation under light-emitting diodes (LEDs) and visible light also induced good bacterial toxicity (Figure 7).

**Figure 8:**
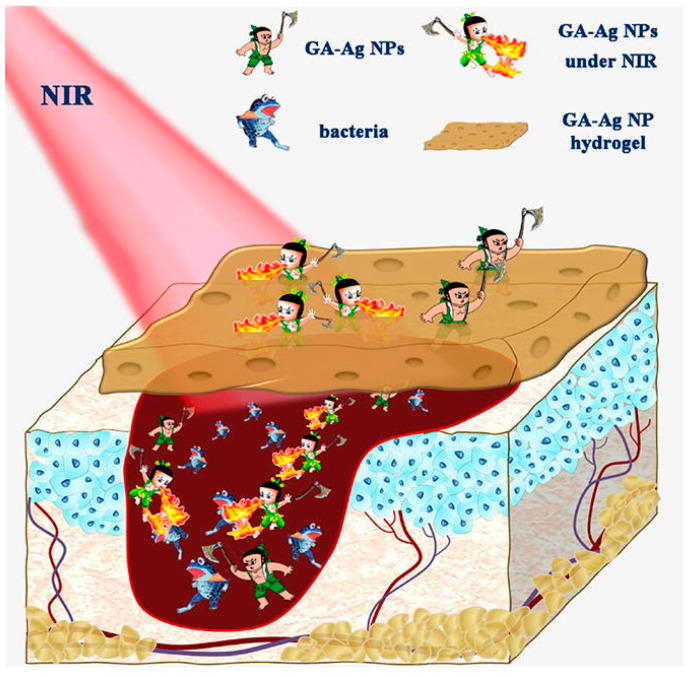
Schematic representation of the photothermal mechanism of antibacterial activity by gallic acid-conjugated silver nanoparticles (GA-Ag NPs). Reproduced from [61], with permission from Elsevier, 2020.

The photothermal conversion efficiency of gallic acid-conjugated silver (GA-Ag) NPs was found to be 48.70%, which is higher than that of other nanocomposites, such as PEG-conjugated MoS_2_ nanoflowers, CuS nanodots, bismuth selenide nanospherical sponges, platinum NPs, and Prussian blue nanocages [61,62]. As previously mentioned, photothermal conversion was higher for GA-Ag NPs than other nanocomposites, making it an effective candidate against antibiotic-resistant microbes. This composite exhibited the effective killing of harmful disease-causing microbes with a swift increase in temperature and the release of silver ions. GA-Ag NPs embedded into carrageenan hydrogels not only exhibited an improved antibacterial effect with 808-nm NIR laser irradiation, but also displayed better wound healing with a photothermal effect in an in vivo rat model. The hydrogels also enhanced the absorbance of NIR radiation by silver ions and assisted with the controlled and speedy release of silver ions. The heat released by GA-Ag NP hydrogels was found to be sufficient to eradicate pathogenic bacteria known to cause vicious infections, and the heat released by these NPs promoted a rapid wound-healing effect. GA-Ag NP hydrogels were found to be effective agents against harmful disease-causing microbes, as well as in speedy wound recovery (Figure 8). GA-Ag NP hydrogels were recently reported to be an effective antimicrobial agent under NIR laser irradiation compared to GA-Ag NP hydrogels alone. NIR laser irradiation at 2 W/cm^2^ for 10 min eliminated up to 98.7% and 94.8% of the microbes *E. coli* and *S. aureus,* respectively. An in vitro bacterial culture also indicated similar results.

There are some examples which are correlated with the present study of GA-Ag NP hydrogels; in the presence of NIR laser radiation, copper sulfide NPs combined with bovine serum albumin (BSA-CuS) nanocomposites showed an enhanced efficiency of photothermal conversion and photothermal effects leading to the elimination of antibiotic-resistant GPB and GNB [63,64]. *N*,*N*′-Di-sec-butyl-*N*,*N*′-dinitroso-1,4-phenylenediamine (BNN6) conjugated with MoS_2_ nanovehicles also exhibited an enhanced killing of bacteria under the influence of NIR laser irradiation [65]. Degradation of the microbial cell envelope was elevated with an increase in temperature caused by GA-Ag NPs under NIR laser irradiation. Microbes were labeled with fluorescent markers, i.e., green fluorescence for live bacterial cells and red fluorescence for dead bacterial cells, and viewed with confocal laser scanning microscopy (CLSM). CLSM images revealed GA-Ag NP hydrogels under NIR laser irradiation compared to GA-Ag NPs alone. In vivo studies demonstrated improved wound healing at 7 days with GA-Ag NPs under NIR laser irradiation. SEM images also indicated similar results, as effective microbial cell wall degradation was observed with GA-Ag NP hydrogels combined with NIR laser irradiation. The heat generated by the photothermal effect led to protein coagulation and degradation of the cell envelope. In that study, the broad-spectrum infection carriers, *E. coli* and *S. aureus*, were successfully eliminated to the maximum limit [66,67,68,69]. *Staphylococcus aureus*-infected wounds showed better microbial inhibition and wound-healing capability by the seventh day with combined GA-Ag NP hydrogel and NIR laser irradiation treatment. The temperature due to laser irradiation reached 50.6 °C near the wound region from 36.4 °C for GA-Ag NPs, indicating effective microbial inhibition compared to their counterparts. Hematoxylin and eosin (H&E) staining showed an acute inflammatory response due to laser irradiation, and there was accelerated wound healing [61].

**Figure 9:**
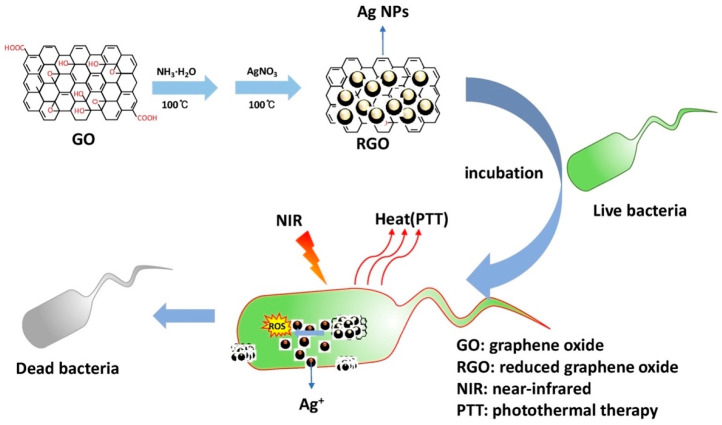
Schematic representation of the photothermal killing of microbes by silver nanoparticles (Ag NPs) embedded in reduced graphene oxide (RGO/Ag). Reproduced from [73], with permission from Elsevier, 2020.

Quaternized chitosan (QCS)-conjugated ferric oxide NPs on the GO surface under NIR laser light displayed a good bacterial inhibitory capability. The photothermal efficacy of chitosan combined with magnetic GO (GO-IO-CS) was found to be effective for microbial inhibition under NIR laser irradiation. Gold nanoshells coated with reduced GO showed a better photothermal conversion efficiency than gold nanoshells or GO alone. Magnetically reduced GO-conjugated glutaraldehyde (MRGOGA) was found to be effective in eradicating ESKAPE pathogens under NIR laser irradiation (10 min), with up to a 99% efficiency. A recent study revealed that Ag-NPs embedded in reduced GO (RGO/Ag) nanocomposites were effective and enhanced inhibition against multidrug-resistant (MDR) bacteria under NIR laser light (at an intensity of 0.30 W/cm^2^) [70,71,72,73]. RGO/Ag nanocomposites were found to be effective antibacterial agents under NIR laser light against ESKAPE pathogens which exhibited antibiotic resistance. In recent times, MDR bacteria possessed by pathogens has become a greater issue, with limited or no solutions. RGO/Ag nanocomposites were found to be effective in tackling this crisis situation. The combination of Ag NPs and RGO increased absorption in the NIR region, which in turn led to an increased photothermal conversion efficiency. The photothermal efficiency of RGO/Ag nanocomposites was found to be better than GO, RGO, or Ag NPs alone. This led to the enhanced photothermal killing of MDR bacteria under NIR light at a power density of 0.30 W/cm^2^ for 10 min (Figure 9). There was alleviation in the temperature showing potential microbial inhibition, with a minimal concentration of RGO/Ag nanocomposites and a low power density of NIR laser light. In that study, two bacteria, *E. coli* and *K. pneumoniae,* displayed respective minimum inhibitory concentrations (MICs) of 15 and 30 μg/mL under 808-nm laser light, and the antibacterial efficiencies were found to be 98.2% and 97.6%. RGO/Ag nanocomposites were effective photothermal agents, and the RGO coating prolonged the release of silver ions. NIR laser radiation for 10 min showed half-MICs for *E. coli* and *K. pneumonia* of 9 and 17 μg/mL, respectively. RGO/Ag is believed to be the most effective photothermal antibacterial agent at the present time when compared to GO, RGO, or Ag NPs alone. Two fluorescent nucleic acids, 4′,6-diamidino-2-phenylindole (DAPI) and propidium iodide (PI), were used to stain *E. coli* cells. Fluorescent images also showed enhanced antibacterial activity under NIR laser irradiation for 10 min. There was irreparable damage caused to bacterial cell envelopes under NIR laser irradiation, as confirmed by fluorescence images [74,75,76,77].

**Figure 10:**
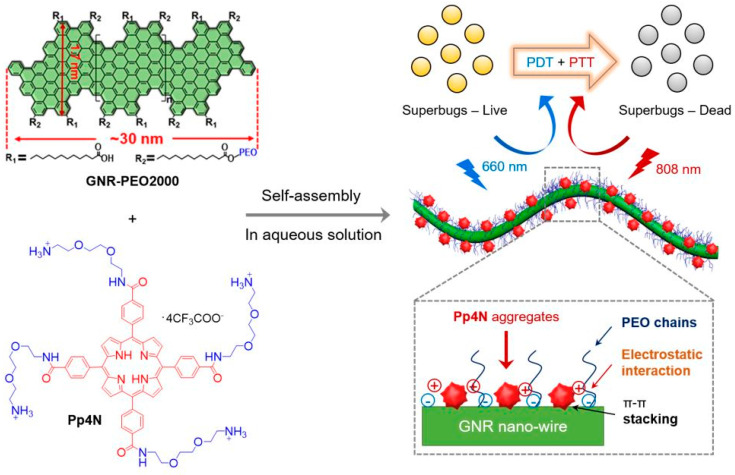
Structures of cationic porphyrin (Pp4N) and water-dispersible graphene nanoribbons (GNRs) with a poly(ethylene oxide) (PEO) grafting percentage of 42% (GNR-PEO2000) used for self-assembly and double-light activated photodynamic and photothermal therapy of drug-resistant bacteria (superbugs). Reproduced from [78], with permission from John Wiley and Sons, 2020.

A nanocomposite from graphene nanoribbons (GNRs) and polycationic porphyrin (Pp4N) was fabricated by Yu and coworkers using a simple self-assembly method [78]. Due to the hydrophobic character of graphene, GNRs were conjugated with poly(ethylene oxide) (PEO) to obtain the hydrophilic form (GNR-PEO2000). GNR-PEO2000 carries a negative charge, Pp4N carries a positive charge, and the composite Pp4N/GNR was obtained by electrostatic attraction using sonication. Upon 808-nm laser irradiation (at 1 W/cm^2^), GNRs induced a time-dependent temperature increase, reaching 53 °C in 10 min, which was similar to that caused by Pp4N/GNR-PEO2000 nanocomposites. More interestingly, GNRs turned out to be a capture agent for GNB and GPB. Under 808-nm laser irradiation, the composite reduced the bacterial viability to 30.8%. When NIR laser irradiation was followed by visible wavelength irradiation, the bacterial viability decreased to 14.3% (Figure 10).

## 4. Polymer-Based Nanomaterials

Hydrogels present multiple advantages for wound dressings, such as oxygenation, water permeability, the preservation of a moist microenvironment, etc. [79,80,81,82,83]. Inorganic nanomaterials such as metals [84], transition metals [85], and carbon-based nanomaterials [86] with a photothermal effect can be incorporated into hydrogels to improve the photothermal capacities. Although inorganic nanomaterials are better photothermal agents, organic nanomaterials also exhibit the same properties. Therefore, organic compound-doped hydrogels were used as photothermal antimicrobials [87,88] and have the advantage of being cost-effective.

**Figure 11:**
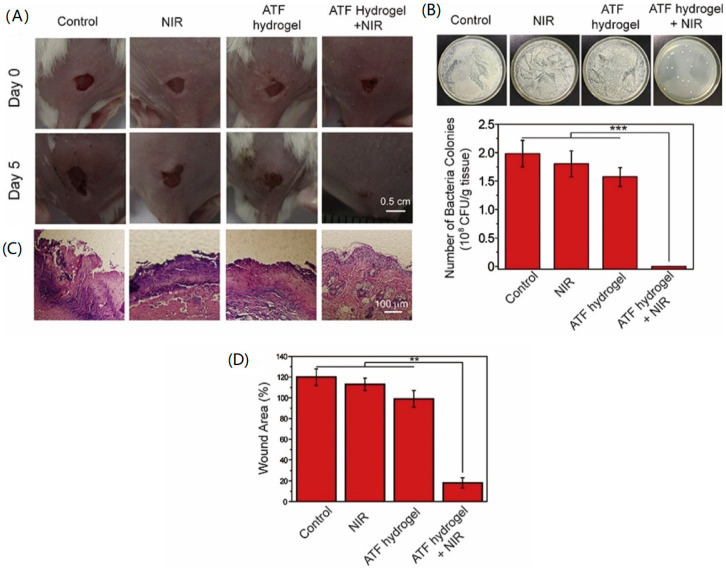
(**A**) Digital images of *S. aureus*-infected wounds of mice before treatment and after different treatments on day 5. (**B**) Digital images and histograms of *S. aureus* colonies derived from homogenized dispersions of the wounds of mice with different treatments, *** *p* < 0.001. (**C**) Digital images of hematoxylin and eosin (H&E)-stained wound sections in mice at day 5 with different treatments. (**D**) The corresponding statistical diagram of wound area healing rates of mice in (**A**). Data are presented as the mean ± SD from six parallel experiments per group (*n* = 6). ** *p* < 0.01. Reproduced from [92], with permission from Elsevier, 2020.

Based on the photothermal effect of tannic acid-Fe (Ta-Fe) [89], and favorable properties of agarose (AG) for biological applications, such as being thermoresponsive and biocompatible [90,91], Deng et al. synthesized Ta-Fe composites (ATFs). ATFs revealed wide absorption in NIR wavelengths of 500~900 nm. Therefore, upon 808-nm laser irradiation, the temperature of ATF increased and was dependent on the laser intensity and irradiation time [92]. After NIR irradiation for 10 min, followed by a bacteriostatic ring test, ATF hydrogels induced an inhibition zone diameter of 15 mm with *S. aureus.* Wounds infected with *S. aureus*, treated with ATF hydrogels, and irradiated exhibited the occurrence of scabs and less edema. The number of colonies after quantification revealed a significant decrease compared to the control group (Figure 11).

**Figure 12:**
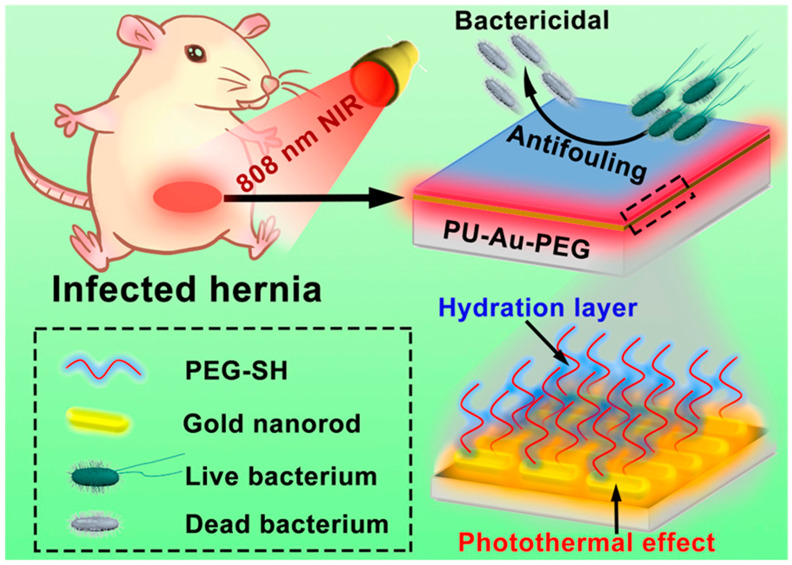
Antibacterial activity of polymeric gold nanorods due to a photothermal effect. Reproduced from [96], with permission from American Chemical Society, 2020.

Huang et al. reported that ferric oxide combined with gold nanoeggs functionalized with vancomycin displayed extraordinary antibacterial activity under NIR light irradiation, and there was a significant temperature increase of 32 °C in 3 min. Polyaniline combined with glycol chitosan NPs was found to be effective against disease-causing microbes under NIR laser light irradiation. Wei Qian and co-workers showed that glycol chitosan combined with carboxyl graphene was better against disease-causing methicillin-resistant *S. aureus* (MRSA) when treated with an NIR laser, and with a controlled pH, speedy wound recovery was observed [93,94,95]. Polymeric nanomaterials were found to be effective in recent studies; polyurethane-conjugated gold nanorods coated with PEG (PU-Au-PEG) nanocomposites were effective anti-infective materials under 808-nm NIR light [96]. PU-Au-PEG nanocomposites were found to be effective against both GNB and GPB under 808-nm NIR light. Gold nanorods possessed a better photothermal efficacy. The photothermal efficiency of gold nanorods was found to have increased when conjugated with PEG and polyurethane under NIR light irradiation. In that work, in vitro and in vivo studies showed the efficient killing of pathogens under NIR light by PU-Au-PEG nanocoatings. Polymer nanocoatings on gold nanorods also demonstrated a better eradication of bacteria when tested with biofilms under NIR light. CLSM images showed the effective killing of disease-causing microbes under NIR light for 10 min. With PU-Au-PEG nanocomposites under NIR light irradiation for 10 min, the temperature increased from 20 to 55 °C, revealing a better photothermal efficiency than other components used alone. In that study, two pathogens, *P. aeruginosa* and *S. aureus,* which are ESKAPE pathogens, were successfully eliminated with 10 min of NIR irradiation. The study showed high bacterial elimination efficiencies due to the photothermal effect (Figure 12). The results were supported by the better antibacterial activities exhibited by these nanocomposites, and the polymers used in the work did not allow microbial adhesion. The study revealed improved antimicrobial activity supported by in vitro and in vivo studies. The antimicrobial activity was observed to only be effective with irradiation using 808-nm light. The PU-Au-PEG nanomaterial used was active under NIR light, biocompatible, and stable, and exhibited an enhanced killing of bacteria [97,98,99,100,101,102,103,104].

**Figure 13:**
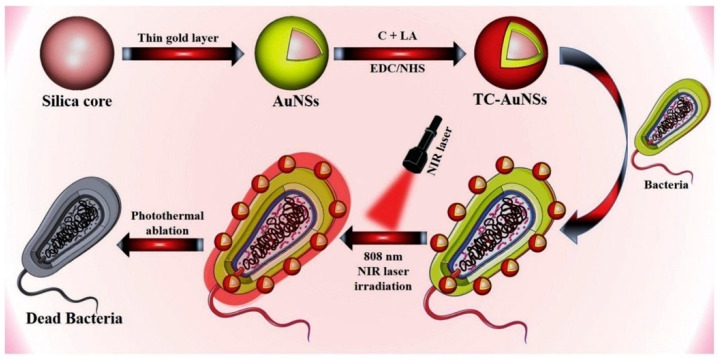
General representation of the photothermal mechanism for bacteria. Reproduced from [106], with permission from Elsevier, 2019.

Dithiols are known to have a greater compatibility with Au NPs. When dithiols are conjugated with Au NPs, the biocompatibility and stability of the latter are increased. Thiol-chitosan gold nanoshells (TC-AuNSs) were reported to display an enhanced bactericidal effect due to the photothermal effect. The thin layer of gold coating over the silica core and conjugation with thiol-chitosan for enhanced stability and application of these gold nanoshells to the bacteria and NIR irradiation impacted the photothermal killing of bacteria (Figure 13) [105,106]. The photothermal efficiency was comparatively high for TC-AuNSs, and the photothermal efficiency was positively affected by an increase in the concentration of TC-AuNSs and the intensity of NIR radiation. The increased concentration of TC-AuNSs and laser radiation intensity were directly proportional to a swift increase in temperature (enhanced photothermal effect) and upgraded antibacterial activity. The combined ability of TC-AuNSs and NIR laser irradiation caused irreparable harm to the cell walls of microbes (Figure 13) [106,107,108,109,110]. Human pathogenic bacteria such as *S. aureus*, *P. aeruginosa,* and *E. coli* are disease-causing agents because of their increased antibiotic resistance. TC-AuNSs were found to be very effective in eliminating these harmful microbes. Using PTT, gold nanostars (GNSs) displayed an excellent antibacterial efficiency of up to 99.99% [111]. Antibiotic-loaded gold nano-constructs demonstrated an enhanced antimicrobial resistance against MRSA with PTT, throwing light on a new approach to deal with antibiotic-resistant bacteria [112].

In recent studies, the oligomer, OF GREEN N, has shown an enhanced photothermal efficiency of 37.7%. The OF GREEN N oligomer displayed enhanced antibacterial activity against *S. aureus* under NIR laser irradiation [113]. Pegylated and thiolated (PEG-SH) gold nanorods (AuNRs) conjugated with a phospholipid (DSPE-AuNR-PEG-SH) and cholesterol (Chol-AuNR-PEG-SH) exhibited an antibacterial effect. The DSPE-AuNR-PEG-SH with NIR light irradiation did not demonstrate a significant increase in temperature. However, the Chol-AuNR-PEG-SH showed a significant increase in temperature of 19.7 °C at 15 min under NIR light irradiation. The Chol-AuNR-PEG-SH displayed a bactericidal effect under NIR light irradiation against one of the ESKAPE pathogens. For obtaining a better photothermal efficiency, there should be adequate adhesion of the NPs to the target cell, tissue, or organ, which was found to be significantly low in the case of DSPE-AuNR-PEG-SH. The Chol-AuNR-PEG-SH was found to exhibit antibacterial activity, perhaps because there was significant adhesion of the NPs to the target skin tissue under NIR light irradiation [114,115].

**Figure 14:**
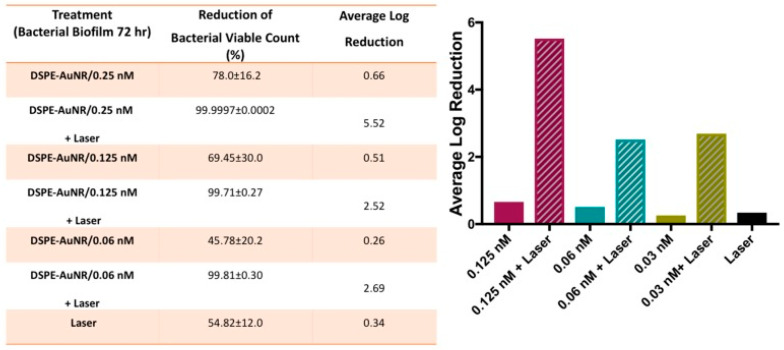
Antibacterial activity under NIR laser light by a phospholipid-conjugated gold nanorods (DSPE-AuNR) suspension loaded into a poloxamer 407 hydrogel against *P. aeruginosa* biofilm (72 h) using a continuous wave (CW) or pulsed laser at 3 and 1 W/cm^2^. (Left) Calculation of the percentage reductions in viable bacteria counts with AuNR/hydrogel after NIR laser excitation (CW vs. pulsed) and in dark conditions. (Right) Calculation of the average log reductions of viable bacterial counts after treatment with AuNR/hydrogel and NIR laser excitation (CW vs. pulsed) and under dark conditions. Reproduced from [116], with permission from MDPI, 2019.

Recently, there have been studies conducted to counter resistant biofilms using hydrogels and phospholipid-conjugated gold nanorods (DSPE-AuNR/hydrogels). In one study, researchers compared pulsed and continuous NIR laser beams on DSPE-AuNR/hydrogels for 15 min. The percentage and average log reductions of viable bacteria counts were calculated. The DSPE-AuNR/hydrogel was found to be more effective against *P. aeruginosa* biofilms incubated for 72 h and treated with pulsed NIR beams for 15 min (Figure 14). The pulsed NIR laser would be less cell destructive and cause less harm to normal, surrounding cells. The bacterial biofilm used in that study was photothermally deactivated with a temperature increase of 50~80 °C for more than 1 h. The DSPE-GNR/hydrogel showed enhanced antibacterial activity under pulsed NIR irradiation for 15 min. Additionally, the antibacterial activity of this polymer-based nanomaterial was confirmed by transmission electronic microscopy (TEM) and fluorescent microscopy [116,117,118,119,120,121,122,123,124,125].

In this review, for investigating the optical properties of nanomaterials, it can be seen that metal-, carbon-, and polymer-based nanomaterials have been designed to absorb NIR light, and NIR light energy has then been transformed into heat for the photothermal killing of bacteria. Moreover, in terms of the photothermal efficiencies of nanomaterials, the solutions of metal-, carbon-, and polymer-based nanomaterials have shown capabilities with respect to increases of their temperatures, including 46, 40, and 58 °C, respectively. The solutions of metal-, carbon-, and polymer-based nanomaterials can be separately heated to 65.3, 60, and 78 °C. Previous studies have reported that NIR light-induced photothermal therapy generally requires hyperthermic temperatures (>43 °C) for the effective killing of bacteria or cancer cells [126]. In sum, the photothermal efficiencies of nanomaterials selected in this review have exhibited superior antibacterial activities.

## 5. Discussion

We have summarized papers with a detailed introduction on treatments of bacterial focal infections utilizing PTT by nanomaterials in this review (Table 1). The works we have described have demonstrated the ability of different nanomaterials to kill bacteria by up to 99.99% in vitro and in vivo with photothermal techniques. As a general trend, the photothermal antibacterial effects of composites of metal, carbon, and polymers turned out to be better than those of the same materials used alone. In addition to generating heat, the composites can provide certain properties, such as enzymatic activity (DNase, protease), ROS generation, enhanced ion release (silver ions), and electrostatic attraction between surface charges of the composite and cell wall charges of the bacteria. These properties are favorable for bacterial destruction and improve the antibacterial effect when they are associated with PTT. Another perspective to encourage in PTT research for bacterial killing is the combination of PTT with other techniques, namely photodynamic and gene therapies, for producing synergistic antibacterial effects. The combination of PTT with different techniques can also avoid the use of very high temperatures, which is toxic to surrounding cells and remains one of the important challenges of PTT.

## Figures and Tables

**Table 1:** Comparison of nanomaterials with photothermal effects as antibacterial agents.

Nanomaterial Types	Nanomaterials and Sizes	Antibacterial Mechanisms	Laser Wavelengths Laser Intensities Irradiation Time	Bacterial Strains	In Vitro and In Vivo	Ref.
Metal-based nanomaterials	AuNWs 5 ± 1.5 nm	PTT	808 nm 1 W/cm^2^ 20 min	*E. coli* *S. aureus*	In vitro	[39]
	PGs 32 × 7.8 nm	PTT and protease activity	808 nm 2 W/ cm^2^ 20 min	*E. coli* *S. aureus*	In vitro	[40]
	MPPI NSs 100∼500 nm	PTT and affinity of bacteria and nanomaterial	785 nm 0.58 W/cm^2^ 10 min	*S. aureus* *P. aeruginosa*	In vitro and in vivo	[42]
	Au-Ag@SiO_2_ NCs ∼155 nm	PTT and silver ion release	808 nm 1 and 1.5 W/cm^2^ 5 min	*E. coli* *S. aureus*	In vitro and in vivo	[46]
	Ti-GNRs surface 49 ± 4 × 11 ± 2 nm	PTT	808 nm 0.5 W/cm^2^ 20 min	*E. coli* *P. aeruginosa* *S. aureus* and *S. epidermidis*	In vitro	[52]
	DNase-AuNCs 2.33 ± 0.72 nm	PTT and PDT and DNase activity	808 nm 2 W/cm^2^ 10 min	MDR GPB and GNB	In vitro	[59]
Carbon-based nanomaterials	RP/GO film 0.65 μm	PTT	SSL and 808 nm 0.2 and 0.6 W/cm^2^ 20 min	*E. coli* *S. aureus*	In vitro	[60]
	GA-Ag NPs 43.1 nm	PTT	808 nm 2 W/cm^2^ 10 min	*E. coli* *S. aureus*	In vitro and in vivo	[61]
	RGO/Ag 58.94 ± 12.30 nm	PTT	808 nm 0.30 W/cm^2^ 10 min	*E. coli* *K. pneumoniae*	In vitro	[73]
	GNRs 45 nm in diameter	PTT and PDT	808 and 666 nm 1 W/cm^2^ 10 min	GNB and GPB	In vitro and in vivo	[78]
Polymer-based nanomaterials	ATF hydrogel 30 × 4 mm	PTT	808 nm 1 W/cm^2^ 10 min	*S. aureus*	In vitro and in vivo	[92]
	PU-Au-PEG 40 × 10 nm	PTT andpolymer bed	808 nm 1.2 W/cm^2^ 3 min	*P. aeruginosa* *S. aureus*	In vitro and in vivo	[96]
	TC-AuNSs Average diameter of 120 nm	PTT	808 nm 0.95W/cm^2^ 5 min	*S. aureus*, *P. aeruginosa*	In vitro	[106]
	DSPE/AuNR 49.8 ± 2.6 × 11.8 ± 1.8 nm	PTT	808 nm 3 W/cm^2^ 15 min	*P. aeruginosa*	In vitro	[116]

Abbreviations: AuNWs: gold nanoworms; PGs: protease-conjugated gold nanorods; MPPI NSs: MoS_2_-coated PDA, PEG-SH, and IgG nanosheets; Au-Ag@SiO_2_ NCs: silica-coated gold-silver nanocages; Ti-GNRs: gold nanorods coated on titanium; DNase-AuNCs: gold nanoclusters coated with DNase; RP/GO: graphene oxide coated on red phosphorous; GA-Ag NPs: gallic acid-conjugated silver nanoparticles; RGO/Ag: silver nanoparticles embedded in reduced graphene oxide; GNRs; graphene nanoribbons; ATF: Ta-Fe composite; PU-Au-PEG: polyurethane-conjugated gold nanorods coated with PEG (PU-Au-PEG); TC-AuNSs: thiol-chitosan gold nanoshells; DSPE/AuNR/hydrogel: hydrogel and phospholipid-conjugated gold nanorod; PTT: photothermal therapy; PDT: photodynamic therapy; SSL: simulated sunlight; MDR: multidrug-resistant; GPB: gram-positive bacteria; GNB: gram-negative bacteria.
